# The Physiological Therapeutics of Religious Mania

**Published:** 1901-08

**Authors:** C. A. F. Lindorme

**Affiliations:** Atlanta, Ga.


### THE PHYSIOLOGICAL THERAPEUTICS OF RELIGIOUS MANIA.

By C. A. F. LINDORME, PH D., M.D.,
ATLANTA, GA.

There is a noteworthy ironical suggestion in the current terminology: If there be a mania which is religious, it follows that it is in the nature of such religion to savor madness; the mania is the normal condition in a paroxysmal exhibit.

This aspect is not changed by a greater analytical closeness. It may be inferred even, that the religious maniac is more religious than the common religious individual. He differs from the latter not in displaying his corrupt personality by deviating from the properly so-called field of religion, but, on the contrary, by cultivating the same in a degree of raging exorbitance. When on the physical field a lunatic insists on the caprice of being the fortunate inventor of a mechanism that runs without any special supply from a specific seat of motor-power, and unmodified in its force-effect by friction, dead weight, etc., or an aeronautic apparatus at the command of the navigator without any locomotion and steerage independent from the climatic agencies, the lunacy explodes on the outer-scientific regions of mentality, in dead contradiction to the established and practically ruling principles. In religious mania this is not so. Here theoretically the lunatic remains within the bounds of the legally reputed sound thinking. But practically he extravagates. He makes an application of the doctrines to his own person which is not customary, legally not admitted sane. So, the cases are not rare that a religious maniac believes himself the Lord, creator of the universe, or Christ, his son. But in the promiscuous reading of half a century I never came across a case where a religious maniac believed himself the uncle or the brother-in-law of the Lord. The regular stock of imagination did not afford a pattern to go by.

Again, as to the degree of craziness in the alienation it cannot be said of the religious maniac that he extravagates beyond the limits of what legally is reputed sane, for the record as to wild encroachments upon the field of the scientifically technical impossible in the revelation of St. John is nowhere beat in the annals of psychiatric asylums or medical case-books. All the difference in the utterance of the religious maniac from the doctrine of the legally reputed sane people, with regard to the pathological extreme, is in a personal reference to himself, for reason of which a case is made against him.

Since religion consists more in a professing than in an acting, more in an utterance than a performing, it follows that it exhibits more in language, in words, than in measures, in function, and it is consequently on the field of abstraction where we have to look for an exhibit of the disorder which psychiatrically culminates in religious mania. Again, since religion has exercised from times immemorial a most considerable influence over all other thought, private and social, it is unavoidable that we discover in the current expression of thought in language defects of thinking which occurred in the remotest phases of the history of abstract thought.

Of all the instances which could be cited of such defects of linguistical expression in metaphysics, influenced by religion, there is none of a deeper interest than the most common one of daily usage. It is of the deepest interest because it refers to man. It is the pronoun "I".

In the common usage of the word, not only popularly but also scientifically, there is not the slightest suspicion of a logically slippery ground. In abstract diction it was tacitly admitted that the pronoun is the symbol of the individual, as a totality. But in the loquacious ease of gracious and ungracious application all substantiality of existence is abstracted away, and the resulting confusion has waxed to a degree as to leave it entirely doubtful, whether the theory is an illusionary understanding of a concrete condition, or whether the concrete condition is a fictitious realization of the theory. Man slipped through the fingers of man in the exertion to evolve the thought of his own personality.

A very typical instance of this eliminative process I had with a bank cashier in Florida: "Nothing short of calomel will keep my liver under control," he said. And similarly others will bring their "I" in an antagonism to their own selfhood, which leaves this a mere abstraction, and its constituting parts like a fleet cut adrift from its moorings, every vessel left to itself, without unity, plan and connection. Nay, worse than that, the "I" will fight the organs which constitute its substance as never a despot his servants, and it is the alimentary canal which has to stand the brunt of the battle. There is hardly any part of the organism of the body but has its rest. The heart, even, as has been judiciously remarked by physiologists, has its moment of rest in the intervals between the beats. But the stomach and appendage never are allowed a holiday. On the contrary, when the other parts of the body club for a merriment, it is the stomach upon which is loaded the task, and the gayer the feast the heavier the load of the digestive tract. And not enough that it is pack-horse and sumpter-mule in all sport, it is punished for rebelliousness, when the expert comes with the medicine-box and finds it choked from the esophagus to the anus, not to say anything of the tract from the mesentery to the capillaries of the vena porta, a region which the "busy practitioner" only too often not takes the time to make researches into.

Thus the parts of which man consists were arrayed against man, and the *bellum omnium contra omnes*, so conspicuous in our social relations, where the euphonic translation is fashionable, "free competition," or, in an up-to-date "version," "if you cannot cut anybody else's throat, cut your own," adapts itself to the individual existence, the very personal life. But my Florida bank-cashier, for sure, had got the clue of his philosophy from his family physician, for there is a professional linkage which connects, in a sort of boomerang-extreme, the upshots of forcible intimacy, the glory of a wild sort of excelsior-surgery to kill the I by operating on an organ, and the impenetrability of spurious medicine, called Christian Science, to let die by a dead reference to the pronoun, without absolutely any consideration of its constituting substantiality.

A very queer substitution of the real in this reality! It is a substitution, this time, for which it is not the pharmacist who is responsible.

The funniest part of the story is, however, yet behind. The transcendentalists, in their metaphysics, in wedging in the plank, that man is an abstractum, after many a wild goose hunt, were far from being aware what they were about in their learned pursuit. Up to this date they ignore, indeed, the truth which augured future when Roger Bacon was prominent among the experts of the art of exploration, and which has been victorious since Francis Bacon drew the diagram of its ramparts, the truth, that it is not our oratory with which the intellectual work begins. The transcendentalists ignore, that, whosoever make a beginning, is anteceded by realty; that rant and cant is not creation, and that *all abstraction is a systematical classification of our primary perception through our five senses.* We need only refer to some striking examples of undeniably true instances of classification, to make this obvious to anybody who wants to understand. There are three abstracta which are of tantamount importance in science. This is the *Plant, Animal* and *Man.* Now, then, what is it with the help of which we succeed to distinguish these three classes? It is conspicuously and most clearly *our observation.* This, however, is taking place exclusively with the help of our five senses. We want to see, to hear, to smell, to touch, to taste, as the case may be, in order to know which is which, and consequently our abstraction, or the order in which we put our sensory-perception, is only *our thought about the impressions of our five senses.* We must defy any scholar, philologists or others, to demonstrate any other result of the scientific analysis of the human language. It is the order by classification in which the linguistical accomplishment consists; all the technique of our communication, reciprocally, is in the impartment of the proportions which are occasioned by the classification which we make of the material under survey.

The most crucial test of the classification of our abstract thought is in the correspondence of its distinguishing features. The positive and the negative marks of the antithetic spheres must coincide in the dividing principle, the statement of what man is, positively, be in keeping with the statement of what the plant is not. And it is only for the sake or formal plenitude of diction that we utter the truism; hitherto the exact line of demarkation between plant and animal organism has not yet been found. Still harder to draw is that between animal and man. If we may claim that the dividing line between man and vegetation is, that man is a growth which owns the faculty of self-culture, which obviously is denied to a plant, it is an exceedingly nice question, if there are not instances of participation in the privileges in the animal kingdom.

Again, if a man makes avail of his faculty of abstraction and its adequate linguistical expression, does it follow that nature must lend herself other but approximately to this manœuvre? The words, in their graphic and phonetic definiteness seem to favor such presumption. But what is a word? It is an arbitrary symbol without any scientific weight whatever. Man is called an individual, and it is thought that thereby he is marked out as a compact entity. But in reality there is nothing of the kind. An individual is at best *an extensive group of physiological conditions*, a definition which is corroborated in its generality by the circumstance that it is applicable to an animal or a plant as well.

If an abstraction is made which is not a true abstraction, but a sham, borrowing the final form of linguistical symbolization, the subsequent use of the word in any ratiocination must needs run out in disharmony, if not blooming nonsense. We gave an example in the pronoun I, which, by elimination of its bodily contents, is brought into an antagonism with itself, the corrupting consequences of which are experienced in the leading ranks of the profession even. We will mention another example which, if we are not mistaken, has never been put on record. This is the *devil*-class. The same was not formed according to the observation of life, from which we derive the criterion of such classification, but dictated by phantasmagorical lust and fabulous imagination. It was said by the transcendentalists, who are responsible for the symbol, that the devil is the principle of the bad. Now, this is obviously wrong. It is a classification which wars most decidedly with the classification of common life, homely parlance and scientific language. If the United States could boast of so much purity of official character as hell, we could be proud. If there be a feature which must be called the worst trait of the decay of man, of civic virtue, it is bribery, active and passive. And in the United States we have so much of it that we must call it the rule, nobility and manhood the exception. But in hell the thing is different. All the officials there, from the head-torturer down to the last hand at the brimstone furnace, are absolutely incorruptible. The faithfulness of Satan in the discharge of his infernal duty is simply unexceptional, something the like of which in our country was never heard of. The conscientiousness of the devil presupposes the most irrefragable probity, and consequently hell must be called an institution of disciplinary stamp, and the devil an agent of piousness and utmost serenity of character. Why, if hell formed a part of the United States the thing would be altogether different. If, for instance, Mark Hanna were running it, he would not disappoint a newcomer about the hospitality practiced there by keeping the doors wide open and all visual access gaping, while, no sooner entered, he is delivered to a clawed and horned hobgoblin to exercise his gambols on, but cultivate the party spirit which gathers the birds of a color. He would not, like the devil, cut in his own flesh by administering to each case in the prescribed dose, but put at ease every shy novice by having a messenger-boy ready to show him to some pleasant barmaid in a cool corner and stand a round of summer drinks. "Well, you see," he would say, "sometimes the commissioners come down from heaven to look up the books; but we don't mind that; we find out the days by our detectives, and then some old acclimated toughs, for an extra consideration, crawl into the furnaces to save appearances." So, it is seen, that if the devil is at all a devil, he is beaten "half a dozen lapses of infinity" by a United States senator. And from this we have to infer that the devil of the common classification is no devil at all, no reality, but a sham abstraction, the phantasy of some melodramatic poet, who was too pedantic to understand that the first the devil sees to is to have a good time with his boat crew.

Similarly counterfeit is the abstraction by which is gained the symbol, soul, or spirit. It is an old subterfuge of the transcendentalists, that the soul, or the spirit, is an exhibit of our consciousness which cannot be evolved synthetically from our sensory observation. But this is a mere pretext to excuse the begging of the question. Psychology admits of the warning of Roger Bacon, too, not to begin with empty talk, but first investigate our real surroundings. Now, the soul, or spirit, is a reality, no doubt about it. But not, as the transcendentalists aver, a dead compactness of unqualified, solid uniformity, which can be numbered like the stalls of a cattle-show, but in agreement to the variegated condition of things which we find in the spiritual man as well as in the bodily one. Just as in the exploration of the bodily man, it is by investigation of his various parts, both regions, outward surface and inner organs, that we attain complete knowledge of the same, it is in the investigation of the spiritual man by exploration of the manifold exhibit of the spirit. Any totality of the spirit of man we can tell only by knowing its parts, and richness or poorness of spirit is constituted by either abundance or scantity of parts. In the detail of our outward investigation we discover the effect of the functionating of the spirit; we speak of the spirit of a horse, and designate therewith qualities which show in the bearing of the animal, but cannot be exemplified in themselves. Consciousness of the soul as such man has only in his self-consciousness, and here again, even, only by becoming aware of the single stirrings of the soul, or spirit. There is in man the spirit of courage and of cowardice, of thrift and lavishness, of generosity and littleness, of faith and jealousy, of adaptation and awkwardness, of pusillanimity and of temerarity, of constancy and fickleness, etc., etc., and a person who lacks any of these sides of human self-assertion can obtain a very defective knowledge only of the respective stirring, while a playwright like Shakespeare cannot be thought but with an altogether exceptional spiritual endowment which enables him to feel as well like the most abject outcast as like the most innocent, guileless virgin.

It was said by Moleschott, "Man is what he eats." We do not want to discuss this here. But thus much is certain, man *is* his stomach, his liver, his alimentary tract and the rest of his organs. Again, his "I" is not consummate, not complete, without the soul which he may claim, because of the special stirrings in him, the totality of which it is which we call soul. There is in a man a spirit of love and of hate, of appetite, striving, and of dullness and satiety, which constitute the greater moiety of his life, and to deny which would be denying life. But to do as the transcendentalists do, make out of the word which designs a class of reality observed a new reality, altogether fictitious, is a puerility; it is as silly as to travel to Washington, D. C., and look in the city directory for the address of Uncle Sam.

If the formation of words, the construing of thought, or the function of abstraction was discharged properly; if, accordingly, in thought due proportion was kept to the sensory conditions and the use of language from which all abstraction must derive, the hotbed of insanity would be avoided where to-day the root of its rank growth in ever increasing dimension is so appallingly fomented. Religious Mania, more particularly, would be a matter of the past: Whatever of positive incitement to craziness would be in the blood, the habit of correct thought established through careful discipline of the brain would act as corrective, and insanity never assume a more severe form than eccentricity, something which there is great interest not to wish entirely away.

What, in observing the proper method of the scientific process, our abstraction is subservient to is the co - and sub-ordination of the detail of our senory knowledge, all the minute items of the nervous impressions. What, as a fact, it mostly tends to in reality, is, to push people of little bias towards eccentricity and land tolerably strong intellects, superior intellects, even, right in the "jaws of ruin and codille." Over the abstracta, eternity, infinity, immortality, many a handicapped intellect went raving mad, which could not have been troubled if properly guided by scientifically plain methodology. The congruence of the words is perfectly adequate if applied only where they belong. The expressions do not refer to bodily dimensions. The sphere they occupy is the soul-life, the spiritual faculty. Immortal is our soul, because by soul we understand the qualifications of our organism which do not exhibit in a specific form, although it is they which do the forming. Infinite and eternal is our being with reference to these qualifications, because they outlast all form and are active in every form and independently of the same. Energy is a mode of exhibit of the spirit, so is love, hate, strength and weakness. Now all these emotions, passions, longings and aversions have no form; they are neither square nor oblong, neither brown nor yellow, and have consequently neither end nor beginning. They have no extension; they do not enter at all into space and time; they are endless, or infinite, eternal.

What, however, have the transcendentalists made of the abstracts? A dimension, that is, something which in order to exist must begin and end, and the end of which that is turned towards the observer, must be and is there all the time without an end, so that the proper definition of infinity of the transcendentalists reads, "an extension which has an end only at one end, while the end at the other end is an end without an end." That is enough to upset the astutest lawyer, if he takes it seriously.

Owing to the fictitious reality of the spurious abstraction of the transcendentalists the spirit is given an empty, meaningless, outward existence. In the concrete existence of our observation the spirit and the body are one reality. Never by anything else but the spurious counterfeit abstraction the spirit gained the independence for which it has so long a time been given credit by unmethodical investigators. There is no other independence of the spirit but one through its word. As a word it exists by itself. Otherwise, it is just through its intimacy with the bodily world that it exercises its enormous power.

There is nothing intrinsic in either soul or body, but through their mutuality in the unity of both. The rub is only in the individual to get both into proper harmony, and it is very significative that the greatest praise of the personality of a man is meant if it is said of him, "he has a level head." The first step to do in acquiring it is to check off against the personality *in toto* the parts of which it is made up.

